# Further analysis of the relationship between atmospheric lead emissions and aggressive crime: an ecological study

**DOI:** 10.1186/s12940-018-0354-5

**Published:** 2018-01-25

**Authors:** Mark Patrick Taylor, Miriam K. Forbes, Brian Opeskin, Nick Parr, Bruce P. Lanphear

**Affiliations:** 1 Department of Environmental Sciences, Faculty of Science and Engineering, Sydney, NSW 2019 Australia; 2 Macquarie University Energy and Environmental Contaminants Research Centre, Sydney, NSW 2019 Australia; 3 Centre for Emotional Health, Department of Psychology, Macquarie University, Sydney, NSW 2019 Australia; 4 Faculty of Law, University of Technology Sydney, PO Box 123 Broadway, Ultimo, Sydney, NSW 2007 Australia; 5 Department of Marketing and Management, Faculty of Business and Economics, Macquarie University, Sydney, NSW 2019 Australia; 6 Faculty of Health Sciences, Simon Fraser University, Vancouver, BC Canada

After completing our study of lead and aggressive crime [10], it came to our attention that we might have erroneously combined lead in air data from two neighbouring air quality monitoring stations for the Boolaroo, New South Wales (NSW) suburb. We re-examined the data we initially compiled in 2011/2012, and it does appear that data from two neighbouring sites with overlapping names have been combined, as described below. Importantly, this error does not affect the results or conclusions of our original study.

## Air quality monitoring stations in Boolaroo, NSW

Since the 1970s, air quality data surrounding the former Pasminco Limited’s lead and zinc smelter at Boolaroo, NSW was collected at a number of sites over more than 40 years. In our study, we sought to use the monitoring stations with the longest continuous records available at each of the six suburbs examined [10].

Our initial analyses were based on data provided by the New South Wales Environment Protection Authority (NSW EPA) compiled in their air quality reports. Based on additional data subsequently released by the NSW EPA, it is evident that we had mistakenly combined data from the government site known as ‘Corner of Lakeview and First Streets’ for years 1975–1985 with the industry site known as ‘First Street’ for years 1986–1993.^1^ The mistake arose because we inferred that these related to same monitoring site. Pasminco collected its own data at or immediately adjacent to the government site known as ‘Corner of Lakeview and First Streets’ and named it ‘Lakeview.’

**Fig. 1:**
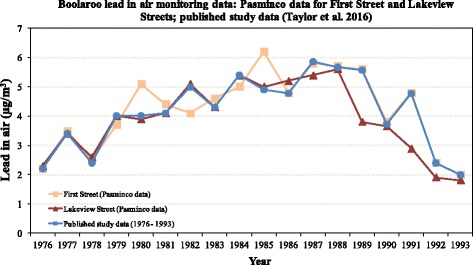
Graphic summary of lead in air data from Boolaroo for the available overlapping years: Published study data [10]; First Street (Pasminco data) and Lakeview Street (Pasminco data). We include the First Street (Pasminco data) because it shows the overlap with our published study data from 1986 onwards

While continuous NSW EPA data are not available beyond 1986 for its ‘Corner of Lakeview and First Streets’ site, Pasminco’s data from ‘Lakeview Street’ are available for the majority of the years assessed in the study (i.e., 1976–1993). We have used these data to re-analyse the results of our study because this data was collected in the residential area of Boolaroo. Lead in air data are also available for the same period of time for Pasminco’s First Street air monitoring site (Fig. 1), which appears to have been located on the company’s land.

## Re-analysis of the data

We initially compared our published data with Pasminco’s Lakeview Street data using two-tailed paired samples *t*-tests. The published data had an average (SD) annual lead level (μg/m^3^) over the study period of 4.1 (1.25), compared to 3.9 (1.23) at Pasminco’s Lakeview Street, and these levels were not significantly different, *t*(17) = 1.582, *p* = .132.

To test whether the error in our data affected our results or conclusions about the relationship between lead in air and aggressive crime, we recalculated the primary suburb-level analyses in two ways: (1) Using the Lakeview Street Pasminco data, and (2) excluding Boolaroo as a site in the analyses.

### Re-analysis with Lakeview street data

Our re-analysis showed that the correlation between lead and crime 21 years later is marginally stronger for Boolaroo using Lakeview Street (Pasminco data) (*r* = .819) compared to our published data (*r* = .802).

Further, re-analysis using the Lakeview Street (Pasminco data) revealed the following:

- The relationship between lead and crime still peaks at a 21 year lag with 38.8% of the variance in assaults accounted for by lead (vs 38.4% in the original published study [10]) with an increase of 200 assaults per 100,000 population (vs 196 assaults per 100,000 population in the original study) for every additional μg/m^3^ of air lead.
- In the full model (including socio-demographic covariates), lead is still the strongest predictor, with a similar ω^2^ (omega-squared) value: 25.8% of the variance in assaults were accounted for by lead using Lakeview Street lead in air measures (vs 26.5% in the original study). We note that there was also a slightly stronger fixed effect (159 vs 154 fewer assaults predicted by a 1 μg/m^3^ drop in lead 21 years earlier).
- The strength, direction, and significance of the socio-demographic covariates in the new model remain unchanged from the initial analysis.

In summary, the statistical analysis and outcomes using the single site Lakeview Street (Pasminco data) are very similar to those in the original study.

### Excluding Boolaroo from the analyses

Excluding the Boolaroo site from the analysis revealed the following:

- The relationship between lead in air and aggressive crime still peaks at a 21 year lag with 32.5% of the variance in assaults accounted for by lead with an increase of 208 assaults per 100,000 population (vs 196 assaults per 100,000 population in the original study) for every additional μg/m^3^ of lead in air.
- In the full model (including socio-demographic covariates), lead remains the strongest predictor, but the results are weaker when the Boolaroo suburb data are excluded (13.4% of the variance in assaults accounted for by lead; 136 fewer assaults predicted by a 1 μg/m^3^ drop in lead 21 years earlier).
- The direction and significance of the effects of the socio-demographic covariates in the new model are unchanged.

In summary, the exclusion of the Boolaroo data from the full model showed that although the effect sizes are somewhat smaller, our conclusions are unchanged (i.e. that lead in air remains the dominant predictor for the lagged shifts in aggressive crime). Thus, overall, we are able to conclude—as we did in the original study—that the results are robust in that there is a strong relationship between lead in air and subsequent rates of aggressive crime.

## Other potential concerns regarding the Boolaroo data

Lead in air monitoring was undertaken at a number of sites in Boolaroo and neighbouring suburbs. In our re-analysis above we also considered whether the use of air monitors close to the lead and zinc smelter may not be representative of community exposures to lead. It was suggested to us that data from the site located at Fourth Street, Boolaroo, would be an ideal community air monitor to use in re-analysis. Unfortunately, data from Fourth Street are only available from the NSW State Pollution Control Commission dating back to 1982 and ceased in 1987 with Pasminco data available from 1988 onwards. Further, our analyses are based on the patterns of covariance between lead and aggressive crime (as opposed to the absolute levels of lead over time alone), and it is these patterns of change over time that form the relationships of interest in our study. Given that the Lakeview Street data captures the patterns of increasing and decreasing levels of lead in air over time in Boolaroo, we are confident in the validity of these data, analyses, and conclusions.

The crime data used in our analyses were based on postcode areas to eliminate the bias introduced by changes in suburb boundaries over the years of interest in the study. The population size of the Boolaroo postcode (c. 10,000) was substantially larger than the population size of the suburb (c. 1000). This mismatch in population size potentially limits interpretation of the effects of lead in air on aggressive crime in Boolaroo in particular (i.e., where this mismatch in postcode versus local area populations was most pronounced). Importantly, as shown above, the conclusions of the study are largely unchanged even when Boolaroo is excluded from the analysis. Given the consistency of the data from the other five suburbs, as well as the state and national data analysed in the original study, we remain confident in the robustness of the findings.

Our findings are consistent with other studies identifying a relationship between childhood lead exposure and the development of antisocial behaviours, including studies unaffected by limitations of ecological data (e.g., [5, 6, 11]). Needleman et al.'s [9] study of bone lead in youths aged 12–18 showed that those exhibiting delinquent behaviours were four times more likely to have bone lead concentrations > 25 ppm than controls. The dose-response effect of lead has also been modelled in rodents, which has shown that animals subject to low level exposures are more likely to exhibit aggressive behaviour (e.g., [4]).

Childhood lead exposure has also been established as a risk factor for conduct disorder [1, 7]. A meta-analysis of lead and conduct disorder involving 8561 children and adolescents concluded lead exposure was problematic for children’s behavioural development [7]. Finally, the National Toxicology Program’s [8] evaluation of the health effects of low-level lead exposures concluded that lead is a risk factor for problem behaviours at blood lead concentrations below 5 μg/dL. The effects of early life lead exposure and the associated behavioural consequences can set a lifelong trajectory of similar behaviours [2, 3], which may be expressed in criminal activities in adulthood. Thus, we conclude, as we had previously, that there is an abundance of data that parallel the findings of our ecological study and show that early life lead exposure is associated with aggressive behaviours, delinquency and related crimes.

The re-analyses of the Taylor et al. [10] data presented here has provided an opportunity to re-consider the original study’s findings. Our findings have further strengthened our confidence in the original conclusions, given the evident robustness of the effects measured.

## Footnotes

1 The NSW State Pollution Control Commission (SPCC) (later morphing into the NSW EPA) ceased monitoring in 1986, after which air quality data was collected by Pasminco and provided to the NSW EPA for reporting purposes.
